# Do children with mental disorders have higher prevalence of hypovitaminosis D?

**DOI:** 10.12688/f1000research.2-159.v1

**Published:** 2013-07-17

**Authors:** Mini Zhang, Keith Cheng, Robert Rope, Elizabeth Martin, Ajit Jetmalani

**Affiliations:** 1 Department of Psychiatry, Oregon Health and Science University, Portland OR, 97239, USA; 2 Department of Internal Medicine, Oregon Health and Science University, Portland OR, 97239, USA; 3 School of Medicine, Oregon Health and Science University, Portland OR, 97239, USA

## Abstract

Inadequate vitamin D level is associated with various adverse medical outcomes. There is a growing concern that insufficient vitamin D may play a role in the development of psychiatric symptoms. This study aims to answer the question: do children with mental disorders have a higher prevalence of hypovitaminosis D? A retrospective chart review examined 25 hydroxyvitamin D (25(OH)D) levels in youth ages 7 to 17 (n=67) at two Oregon psychiatric residential facilities. Vitamin D deficiency is defined as <20 ng/ml and insufficiency as <30 ng/ml. Diagnoses were organized into six categories. 25(OH)D levels were compared across genders and diagnostic groups using a two-sample t-test and ANOVA, respectively. Statistical differences in prevalence across diagnostic categories were calculated using a Pearson chi-square test. Using the data from Saintonge’s NHANES III study on healthy US children for comparison, 21% of our cohorts were found to be vitamin D deficient and 64% insufficient, in contrast to 14% and 48%, respectively. While our results are not statistically significant, mainly because of small sample size, the overall mean 25(OH)D level in our cohort was insufficient (27.59 ± 9.35 ng/ml), compared to a sufficient mean value of 32.1 ng/ml in the general population. No statistical significant difference was found in the prevalence across diagnostic categories. This study found that children with psychiatric disorders might have a higher prevalence of hypovitaminosis D than the general pediatric population. Although a causal relationship between hypovitaminosis D and psychiatric disorders cannot be derived based on the study design, our study provides initial descriptive data on the prevalence of hypovitaminosis D in children with psychiatric disorders, which has not been previously reported to our knowledge. Prospective studies with a larger sample size and controlled variables would allow more precise analysis of the relationship between hypovitaminosis D and childhood mental disorders.

## Introduction

An inadequate vitamin D level is increasingly being linked to diverse disease states. Beyond its importance in endocrine and bone health, there is a growing concern that vitamin D insufficiency may affect brain function and mental health.

Studies have linked hypovitaminosis D to various psychiatric disorders such as depressed mood ^1^ and schizophrenia ^2, 3^. In children, emerging evidence suggests that vitamin D plays a role in brain development ^4– 6^. A Finnish study found that vitamin D supplementation in infancy reduced the risk of schizophrenia later in life among males ^7^. A Swedish study found that patients diagnosed with schizophrenia and autism had the lowest 25-hydroxyvitamin D (25(OH)D) levels among psychiatric diagnoses, and proposed that low 25(OH)D may not only be a predisposing developmental factor, but may also affect mental health in adulthood ^3^. Therefore, prevention of hypovitaminosis D in early life may be associated with reduced risk of developing certain psychiatric disorders.

The optimal level of vitamin D is controversial. The cutoff serum levels range from 20 to 50 ng/ml ^8^. The American Academy of Pediatrics (AAP) recommends a minimal level of 20 ng/ml in children. Using the same cut-off value, the US Institute of Medicine (IOM) reports that the majority of North Americans have sufficient vitamin D required for bone health ^8^. However, some subgroups, particularly those who are older, those living in institutions, or those with darker skin, may be at increased risk for hypovitaminosis D ^8^.

Vitamin D insufficiency in the general population was estimated to range from 1% to 78% among different studies ^9^. Using the Third National Health and Nutrient Examination Survey (NHANES III, 1988–94) data, Saintonge *et al.* estimated that 14% of healthy children had 25(OH)D levels below 20 ng/ml, and 48% had levels below 30 ng/ml ^10^. The prevalence of hypovitaminosis D among children with mental disorders remains unclear.

This study attempts to ascertain whether children with mental illness have a higher prevalence of hypovitaminosis D and whether there is a difference in prevalence across various disorders.

## Methods

A retrospective chart review was conducted at two residential psychiatric treatment programs in Oregon, USA, (latitude 45°N). There were 67 patients aged from 7 to 17 years, whose serum 25(OH)D levels were measured between October 2009 and 2010. Patients had one to four co-morbid psychiatric diagnoses. There were no exclusion criteria. Given the retrospective nature and lack of identifiable health data used in the study, no institutional review board approval was needed.

Deficiency was defined as <20 ng/ml, based on the AAP recommended value. Insufficiency was defined as <30 ng/ml, by the local laboratory standard used in Oregon. For the 14 patients who had multiple 25(OH)D levels recorded, we used the lowest 25(OH)D level in our analyses. We felt this method was justified clinically if any period with this degree of hypovitaminosis D during childhood is correlated to developmental differences. The diagnoses were organized into six categories shown in Table 1. For patients with multiple diagnoses, their 25(OH)D level was counted individually in each diagnostic category to calculate the mean and prevalence.

**Table 1.** Diagnostic categories.

Category	Diagnoses included
Anxiety disorder	• Anxiety Post traumatic stress disorder • Obsessive compulsive disorder • Post traumatic stress disorder
Autism spectrum disorders (ASD)	• Autism • Asperger's syndrome • Pervasive developmental disorder
Disruptive disorders	• Attention deficit/hyperactive disorder (ADD/ADHD) • Conduct disorder • Disruptive disorder not otherwise specified (NOS) • Intermittent explosive disorder • Oppositional defiant disorder
Mood disorders	• Bipolar disorder I and II • Cyclothymia • Major depression
Psychotic disorders	• Psychotic disorder NOS • Schizoaffective disorder • Schizophrenia
Other disorders	• Acculturation problem • Cognitive disorder • Eating disorder • Enuresis • Language/communication disorder • Learning disorder • Relational problem • Sleep disorder • Substance abuse and dependence

25(OH)D levels were compared across genders and diagnostic groups using a two-sample t-test and ANOVA, respectively. Statistical differences in prevalence across diagnostic groups were calculated using a Pearson chi-square test. The analysis was performed using STATA IC (version 11) from StataCorp LP, College Station, Texas.

## Results

A total of 67 patients with 168 diagnoses were included in this study. Using the NHANES III study ^10^ for comparison, 21% of our cohort were vitamin D deficient (<20 ng/ml), compared to 14% reported in the general US population. If a cut-off value of 30 ng/ml was used, 64% of the children in our study population were classified as being insufficient, compared to 48% of healthy US children.

The overall mean 25(OH)D level in our study cohort was 27.59 ± 9.35 ng/ml (i.e. insufficient), compared to a mean value of 32.1 ng/ml (i.e. sufficient) in the general US population ^10^. Females in our study (n=29) had a mean level of 27.4 ± 9.1 ng/ml, comparable to the mean of 27.74 ± 9.66 ng/ml amongst males (n=38). The gender difference was non-significant (p=0.89). The mean 25(OH)D levels by diagnostic category are shown in Table 2. No statistical significant differences could be concluded in the mean level (p=0.80) across diagnostic categories.

**Table 2.** Percent and mean 25(OH)D level by diagnostic categories.

Category	Percent of study cohort (n=168)	Mean 25(OHD) level (ng/ml)	Std. dev. (ng/ml)
Anxiety disorder	18% (n=30)	27.89	10.58
Autism spectrum disorders (ASD)	9% (n=15)	29.72	11.58
Disruptive disorders	27% (n=46)	27.27	6.74
Mood disorders	27% (n=45)	28.96	10.11
Psychotic disorders	4% (n=7)	26.47	12.42
Other disorders	15% (n=25)	26.02	10.85

**Figure 1.**
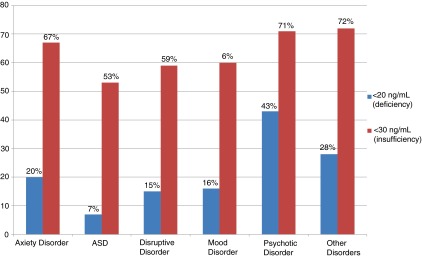
Prevalence of 25(OH)D deficiency (p=0.28) and insufficiency (p=0.72) across diagnostic categories in a pediatric population with mental disorders. Values are given as percentages. See Table 1 for a list of ‘Other Disorders’.

The prevalence of patients with hypovitaminosis D across diagnostic groups using both cutoff values are shown in Figure 1. There was no statistical significance found in the prevalence across diagnostic groups, perhaps due to the small sample size. It is interesting to note that psychotic disorder had the highest prevalence of deficiency and insufficiency among specific diagnostic groups: 43% and 71%, respectively.

Vitamin D levels in a pediatric population with mental disorders at two residential psychiatric treatment programs in Oregon Hypovitaminosis file: 25(OH)D levels (ng/ml) of children with various mental disorders at two residential psychiatric treatment programs in Oregon, USA. Some children had multiple 25(OH)D levels taken. See Table 1 in the main article for disorders that fall under the ‘Other Disorders’. NOS – not otherwise specified.
Vitamin D by diagnostic category file: Some children in the study had multiple mental disorders so here we have arranged vitamin D level (ng/ml) by diagnostic category. See Table 1 in the main article for disorders that fall under the ‘Other’ category. ASD – autistic spectrum disorder.

## Discussion

Beyond its importance in endocrine function, there is growing awareness of the role that vitamin D plays in brain function. However, the prevalence of hypovitaminosis D in children with mental illnesses is uncertain. This study found that children with serious psychiatric disorders may have a higher prevalence of hypovitaminosis D and a lower mean 25(OH)D level, compared to the general US population ^10^. Although no statistical significance can be concluded, it is noteworthy that psychotic disorders had the highest prevalence of hypovitaminosis D among the specific diagnostic categories, which supports previous studies ^2, 3, 7^. We suggest using a cut-off value of 20 ng/ml for clinical interventions, as recommended by the AAP and IOM ^10^. Clinicians should discuss the costs and benefits of treatment with patients when levels are between 20 and 30 ng/ml.

The primary limitation of this study was its small sample size. Due to its retrospective design, we were limited by the availability of 25(OH)D studies without specific clinical indications. Other limitations include not controlling for the length of inpatient stay, ethnicity, age, nutritional status, sun exposure, or skin pigmentation. For the patients with multiple diagnoses, their 25(OH)D was counted in each diagnostic category, which might overestimate the prevalence of hypovitaminosis D. The study utilized the lowest level of 25(OH)D for patients with multiple measurements, which might result in lower mean level and higher prevalence. Although a causal relationship between hypovitaminosis D and psychiatric disorders cannot be derived based on the study design, our study provides important initial descriptive data on the prevalence of hypovitaminosis D in a pediatric population with psychiatric disorders which has not, to our knowledge, been previously reported.

## Conclusion

As research continues on the impact of vitamin D in medicine, its implication for psychiatric disorders may be clarified. While no robust statistical conclusions can be made mainly due to small sample size, this study provides initial data suggesting that children with mental illnesses might have lower vitamin D levels and a higher prevalence of hypovitaminosis D than the general population.

Given the high prevalence of hypovitaminosis D and its profound impact on overall health, clinicians should have a higher suspicion of hypovitaminosis D in the pediatric psychiatric population.

Important future steps include the design of a larger prospective study with more controlled variables would allow more precise analysis to establish the prevalence of hypovitaminosis D, as well as to infer any correlation between hypovitaminosis D and childhood mental illness. Preventative and ameliorative measures might subsequently be instigated to assess causation and affect the development and treatment of certain mental disorders.
