# Supplementary material for: Do children with mental disorders have higher prevalence of hypovitaminosis D?
Source: F1000Res. 2013 Jul 17;2:159. [Version 1] doi: 10.12688/f1000research.2-159.v1 (PMC3886792; doi:10.12688/f1000research.2-159.v1)
Supplement: Peer review history

# Peer review: Referee response for version 1

**Published:** 2013-07-31

**Authors:** Hans-Peter Volz

**Affiliations:** 1 School for Psychiatry and Psychotherapy, Hospital for Psychiatry, Psychotherapy and Psychosomatic Medicine Schloss Werneck, Bavaria, Germany

- The authors should clearly state whether they included all patients of this age group or whether the included patients represent a sub-sample; if yes, according to which inclusion criteria?
- Since patients with multiple diagnoses have been counted in each diagnostic category they fulfilled, a second analysis would be helpful in which every patient is counted only once.
- Was there any medication given that could influence vitamin D-Levels?
- If the authors can meet these points of criticism, the paper is well suited to be approved

I have read this submission. I believe that I have an appropriate level of expertise to confirm that it is of an acceptable scientific standard, however I have significant reservations, as outlined above.

# Peer review: Referee response for version 1

**Published:** 2013-07-25

**Authors:** Linda Mayes

**Affiliations:** 1 Yale University, New Haven CT, USA

While the report offers an interesting hypothesis, the sample is under-powered and the findings are not significant. There are also many biases in why vitamin D levels were or were not measured in psychiatric samples. The authors note limitation of sample size. I would suggest increasing sample size and being more targeted in the definition of psychopathology in future case control design.

I have read this submission. I believe that I have an appropriate level of expertise to confirm that it is of an acceptable scientific standard, however I have significant reservations, as outlined above.

# Response

**Published:** 2013-07-29

**Authors:** Mini Zhang

**Affiliations:** Oregon Health & Science University, USA

Dear Dr. Mayes, 

Thank you for your comments and suggestions. We are aware of several limitations of our study including the sample size and the method of vitamin D measurement, as we stated in our paper. We agree that a bigger sample size and better design with controlled variables, and a prospective study will significantly increase the power and validity of our study. This study is the initial step, and we hope we would be able to pursue this a step further in the future. 

Thank you again for your time and expert opinion. 

Regards, 

Mini Zhang, MD

# Peer review: Referee response for version 1

**Published:** 2013-07-23

**Authors:** Shailesh Jain

**Affiliations:** 1 Texas Tech University, Lubbock TX, USA

The title of the article is appropriate to the content of the article.  

The abstract is able to summarize the key elements of the research including the brief overview of the study design, statistical methods used and the key conclusion. 

The article is well constructed and clear in its presentation. It is succinct, direct, to the point and conveys clear message.

Study design has been conducted, properly measured and collected appropriately. Due diligence has been made as pointed out regarding privacy of the patients. The ethnicity of IRB involvement is also clearly pointed out.

The study design and methodology has been clearly delineated. The methodology and the steps described in this study can easily be replicated in future studies.

The data are presented in an easy to understand and informative way which a busy reader can easily grasp the key findings.

The study presentation has included all the data that are necessary to understand the key findings and helpful for future studies.

Limitations are adequately described though the fact that other possible explanations, such as medical conditions, are not taken into account. 

Though the data ‘look’ okay, I would recommend a review by a statistician.

I have read this submission. I believe that I have an appropriate level of expertise to confirm that it is of an acceptable scientific standard.

# Response

**Published:** 2013-07-25

**Authors:** Mini Zhang

**Affiliations:** Oregon Health & Science University, USA

Dear Dr. Jain, 

Thank you very much for your nice words and comments! We appreciate your time and expertise in reviewing our article. 

Please feel free to contact me if you have further comments or questions. 

Sincerely, 

Mini Zhang, MD
